# Leaf Production and Expansion: A Generalized Response to Drought Stresses from Cells to Whole Leaf Biomass—A Case Study in the Tomato Compound Leaf

**DOI:** 10.3390/plants8100409

**Published:** 2019-10-12

**Authors:** Garance Koch, Gaëlle Rolland, Myriam Dauzat, Alexis Bédiée, Valentina Baldazzi, Nadia Bertin, Yann Guédon, Christine Granier

**Affiliations:** 1 Univ Montpellier, INRA, Montpellier SupAgro, LEPSE, 34095 Montpellier, France; garancekoch@gmail.com (G.K.); gaelle.rolland@inra.fr (G.R.); myriam.dauzat@inra.fr (M.D.); alexis.bediee@inra.fr (A.B.); 2 Unité Plantes et Systèmes de culture Horticoles, INRA, UR 1115 PSH, F-84000 Avignon, France; valentina.baldazzi@inra.fr (V.B.); nadia.bertin@inra.fr (N.B.); 3 Université Côte d’Azur, INRA, CNRS, ISA, 06903 Sophia-Antipolis, France; 4 Université Côte d’Azur, INRIA, INRA, CNRS, Sorbonne Université, BIOCORE Team, 06903 Sophia-Antipolis, France; 5 Univ Montpellier, CIRAD, INRA, Montpellier SupAgro, AGAP, 34095 Montpellier, France; yann.guedon@cirad.fr

**Keywords:** leaf growth, leaf production, cell division, cell expansion, endoreduplication, drought, plasticity, tomato

## Abstract

It is clearly established that there is not a unique response to soil water deficit but that there are as many responses as soil water deficit characteristics: Drought intensity, drought duration, and drought position during plant cycle. For a same soil water deficit, responses can also differ on plant genotype within a same species. In spite of this variability, at least for leaf production and expansion processes, robust tendencies can be extracted from the literature when similar watering regimes are compared. Here, we present response curves and multi-scale dynamics analyses established on tomato plants exposed to different soil water deficit treatments. Results reinforce the trends already observed for other species: Reduction in plant leaf biomass under water stress was due to reduction in individual leaf biomass and areas whereas leaf production and specific leaf area were not affected. The dynamics of leaf expansion was modified both at the leaf and cell scales. Cell division and expansion were reduced by drought treatments as well as the endoreduplication process. Combining response curves analyses together with dynamic analyses of tomato compound leaf growth at different scales not only corroborate results on simple leaf responses to drought but also increases our knowledge on the cellular mechanisms behind leaf growth plasticity.

## 1. Introduction

Natural ecosystems and crops are severely affected by soil water deficit, which has an impact on plant growth and reproduction [1]. The occurrences of soil water deficit are getting increasingly more frequent due to climate changes, and they pose a major threat to the global food security [2]. Recent anthropogenic emissions of greenhouse gasses are the highest in known history, and the risk of severe heat waves and droughts is now particularly high. Nevertheless, soil water deficit is an event that has occurred naturally for centuries, and living organisms have evolved multiple mechanisms to cope with unfavorable environmental conditions such as drought and/or to limit their effects [3]. Depending on drought scenario, species, and/or genotypes, plants tend to adjust their duration of life cycle (drought escape); their water consumption or water status (drought avoidance) to cope with unfavorable soil water content conditions [4]. These different strategies are not exclusive as for example, adjustments of anatomy and/or physiology often reported for drought avoidance or tolerance strategies may also accelerate completion of the life cycle [4]. These behaviors have been well-studied and described when they occur during the plant cycle, but it is also known that species that have encountered drought periods for many generations have developed specialized anatomical features as highly suberized roots, sunken stomata, and thick cuticle layer on their laminas [5].

Whatever the water deficit scenario, these strategies are deployed at the cost of decreased aerial biomass accumulation [6,7,8]. One of the first visible and quantifiable responses to drought at the macroscopic scale is a reduction in whole plant leaf expansion. This response results from a plethora of other responses at different underlying scales, including molecular, biochemical, physiological, and cellular changes. The extent of the decrease in leaf surface depends on the intensity of the drought stress [9,10,11], but also on its timing during leaf development [12]. Drought stresses occurring early during leaf development or during whole leaf development, affect leaf surface with reduction in both cell expansion and cell division [13,14,15,16]. Drought stresses occurring later, only during the second part of leaf expansion, when cell division has ceased, affect leaf surface with a reduction in cell expansion [17]. In a few studies, drought stress scenario did not cause any reduction in cell division, but it is unclear in those studies if the timing of the drought scenario was early enough to affect the phase of cell division within the leaf [18,19]. Furthermore, in species with leaves in which cells endoreduplicate, such as *A. thaliana* or canola, drought stress also affects cell endoreduplication, i.e., a modified cell cycle without mitosis, and reduces cell ploidy [20,21]. Together with changes in leaf surface, some drought scenarios also cause changes in leaf anatomy with increase dry matter content per leaf area, as well as higher epidermal cell and stomatal density [22,23,24].

In most studies considering leaf growth responses to drought, two or three levels of soil water deficit scenario at most are compared, i.e., one control and one or two drought treatments [19,20,25]. Multi-scale analyses are often performed (1) at the final stage of leaf expansion, giving insights into the final result of a dynamic process or (2) at a few stages during leaf development mainly comparing phases with cell division with those without cell division [20]. All previous cited works on leaf growth response to drought have been performed on plants with simple leaves either monocots or dicots. Compound leaves have been less considered, as they seem more complex by their architecture, with leaves composed of successive leaflets attached to the rachis by individual petiolules. Our recent work on tomato leaf development, analyzing leaf growth related traits at the leaflet and cellular scales in successive leaves on a sympodial unit, revealed that compound leaf framework of analysis could be simplified as a leaflet within a leaf resumed the characteristics of this leaf [26].

Taking the benefits of these results, the aim of the present study was to investigate the effects of different drought treatments on tomato leaf growth at different scales, from a whole sympodial unit to sub-cellular scales. We used automatic watering to impose precise drought treatments and imaging techniques for multiscale analyses during leaf expansion. Combining dose response analyses of leaf-related traits together with the dynamics of the underlying cellular traits gave insights into leaf growth responses to drought. Our results corroborated existing data published for the model species *Arabidopsis thaliana* and different crop species with simple leaves, but they also contribute to decipher the respective effects of drought on cell division, cell expansion and endoreduplication in the leaf, revealing precise timing of drought responses on these three processes.

## 2. Results

### 2.1. Tomato Leaf Biomass of the First Sympodial Unit Is Affected by the Different Drought Treatments, Mainly Through a Reduction in Individual Leaf Area and Dry Mass

**Figure 1:**
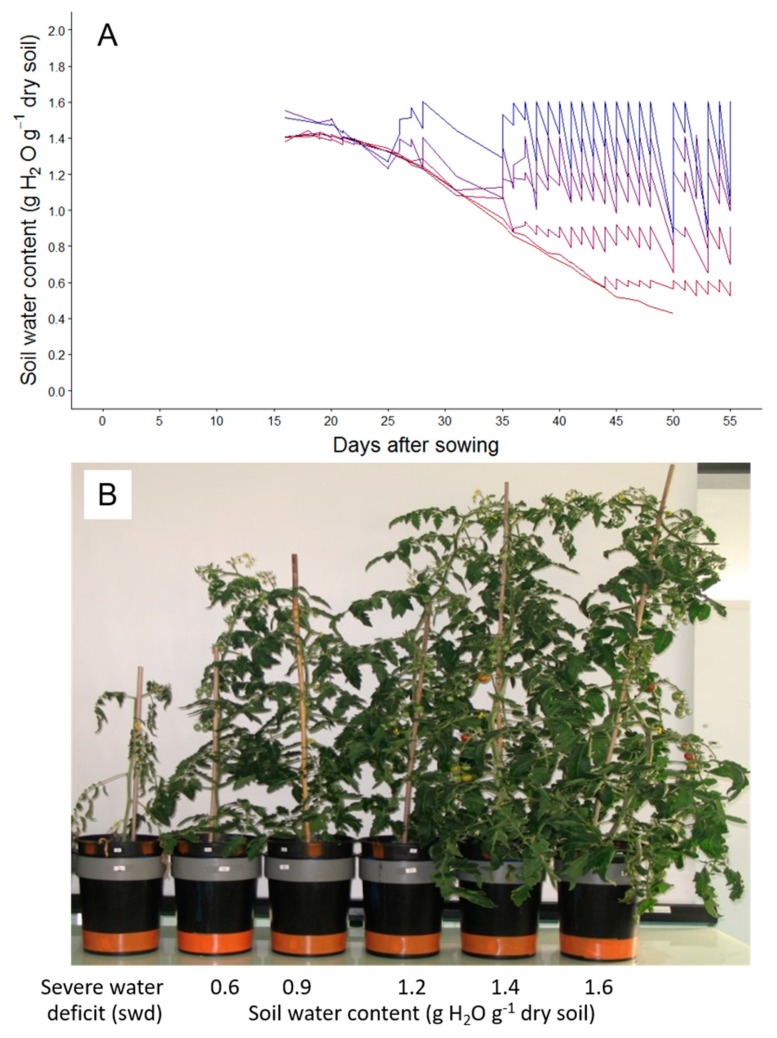
Changes with time in soil water content for the six watering treatments used during experiment A (**A**) and pictures of representative Wva106 plants grown with these six watering treatments 69 days after sowing (**B**). In A, data are means of soil water content calculated before and after daily irrigation for each watering regime, with the PHENOPSIS automaton (*n* = 7, 7 pots in each treatment). In B, Plants are ordered from the driest soil water content on the left to the wettest on the right. The plant on the left corresponds to a plant grown without irrigation after leaf five emergence (severe water deficit (swd)) and the five others to the five watering regimes characterized by the target soil water content when it was stabilized over time (0.6, 0.9, 1.2, 1.4, and 1.6 g H_2_O g^−1^ dry soil respectively from left to right).

As a first step, dose responses between leaf growth, development-related traits, and soil water contents were established to analyze how these traits are affected by drought intensity (Figure 1A,B). For this purpose, plants were subjected to six treatments mainly differing in soil water contents (Figure 1A,B). 

**Figure 2:**
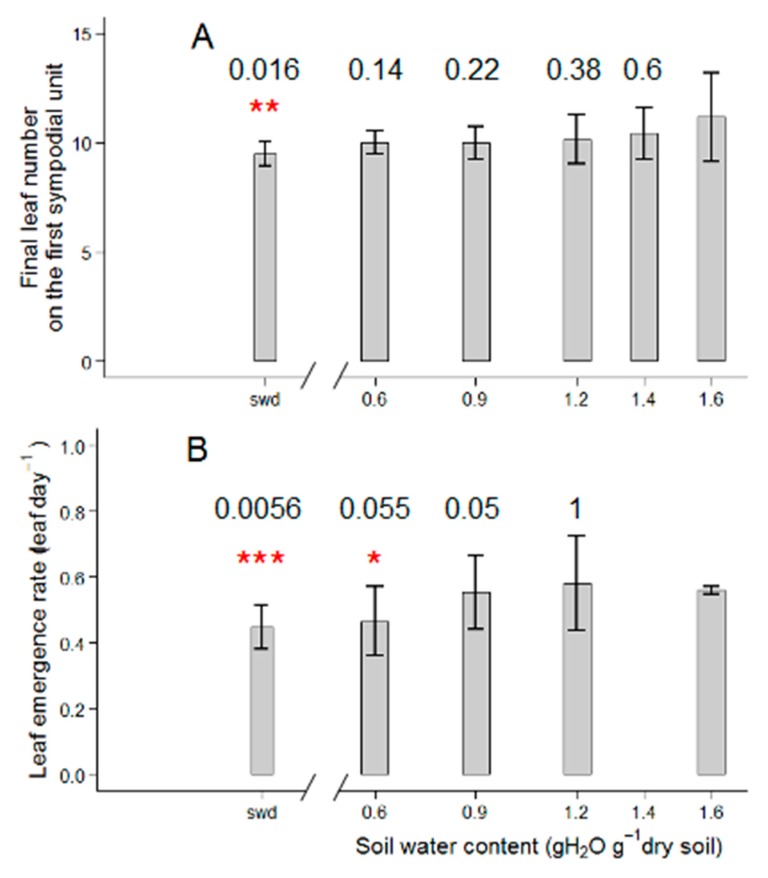
Soil water content response of leaf production-related traits: Final leaf number (**A**) and leaf emergence rate (**B**) in Wva106 plants grown at five different but stable soil water contents: 0.6, 0.9, 1.2, 1.4, and 1.6 g H_2_O g^−1^ dry soil and a severe swd. Means and standard errors are presented by bar charts and vertical lines, respectively (3 *< n* < 7). Leaf emergence rate could not be calculated for the 1.4 g H_2_O g^−1^ dry soil because of typing errors during notations of phenological stages. Significant differences between mean trait values for plants grown at 1.6 g H_2_O g^−1^ dry soil versus other soil water contents are shown by stars above bars according to Wilcoxon-Mann-Whitney statistical test (*p*-value ≤ 0.01 = ***, 0.01 < *p*-value ≤ 0.05 = **, 0.05 < *p*-value ≤ 0.1 = *, *p*-value > 0.1 = null hypothesis cannot be rejected), *p*-values are given above the bars.

**Figure 3:**
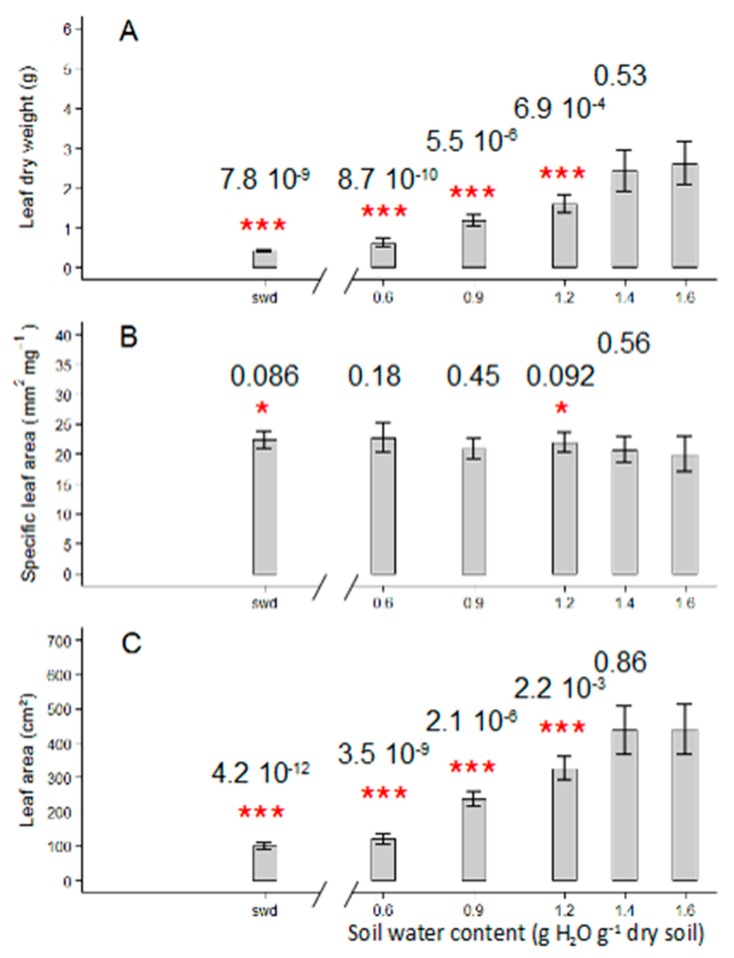
Soil water content response curve of leaf growth-related traits: Leaf dry weight (**A**), specific leaf area (**B**), and leaf area (**C**) in Wva106 plants grown at five different but stable soil water contents: 0.6, 0.9, 1.2, 1.4, and 1.6 g H_2_O g^−1^ dry soil and a severe swd. Data are shown for leaves seven to 10 of the first sympodial unit. Means and standard errors are presented by bar charts and vertical lines, respectively (3 < *n* < 7). Significant differences between mean trait values for plants grown at 1.6 g H_2_O g^−1^ dry soil versus other soil water contents are shown by stars above bars according to Wilcoxon-Mann-Whitney statistical test (*p*-value ≤ 0.01 = ***, 0.01 < *p*-value ≤ 0.05 = **, 0.05 < *p*-value ≤ 0.1 = *, *p*-value > 0.1 = null hypothesis cannot be rejected), *p*-values are given above the bars.

The final leaf number of the first sympodial unit was only slightly reduced by the reduction in soil water content, in a range from 0.6 to 1.6 gH^2^O g^−1^ dry soil (Figure 2A). Wva106 plants had 11.2 leaves when grown at 1.6 g H_2_O g^−1^ dry soil, 10 leaves when grown at 0.6 g H_2_O g^−1^ dry soil and 9.5 leaves when the soil water content was severely reduced without re-irrigation (Figure 2A). This result was consistent with the decrease in leaf emergence rate at low soil water contents compared to the highest ones (Figure 2B). The maximal leaf emergence rate was around 0.67 leaf d^−1^ for plants grown at 1.6, 1.4 and 0.9 g H_2_O g^−1^ dry soil (Figure 2B). It was reduced to 0.47 leaf d^−1^ for plants grown at 0.6 g H_2_O g^−1^ dry soil and was even most reduced to 0.44 leaf d^−1^ under the most severe soil water deficit treatment (Figure 2B). Final individual leaf dry weight, i.e., the mean dry weight of leaves seven to 10 on the first sympodial unit, was maximal for plants grown at 1.6 and 1.4 g H_2_O g^−1^ dry soil (mean final dry weight was 2.62 g and 2.42 g, respectively, Figure 3A). It was reduced by other watering treatments with a minimum value for plants grown in decreased soil water content without re-irrigation (mean final dry weight was 0.42 g per leaf, Figure 3A). Similarly, the final individual leaf area, i.e., the mean final leaf area of leaves seven to 10 on the first sympodial unit, was maximal and reached 440.7 cm² and 438.8 cm² for plants grown at 1.6 and 1.4 g H_2_O g^−1^ dry soil, respectively (Figure 3C). The mean individual leaf area was reduced quasi-proportionally by other scenarios and reached 120.2 cm² for plants grown at 0.6 g H_2_O g^−1^ dry soil (Figure 3C) and 98.9 cm² under the severe soil water deficit treatment (Figure 3C). In contrast, the specific leaf area was not affected by the different watering treatments and was around 21.3 mm² mg^−1^ and a slight increase up to 22.3 mm² mg^−1^, was measured for plants grown at the lowest soil water content (Figure 3B).

### 2.2. Soil Water Deficit Affects the Dynamic of Leaflet Expansion, Epidermal Cell Production and Epidermal Cell Expansion

**Figure 4:**
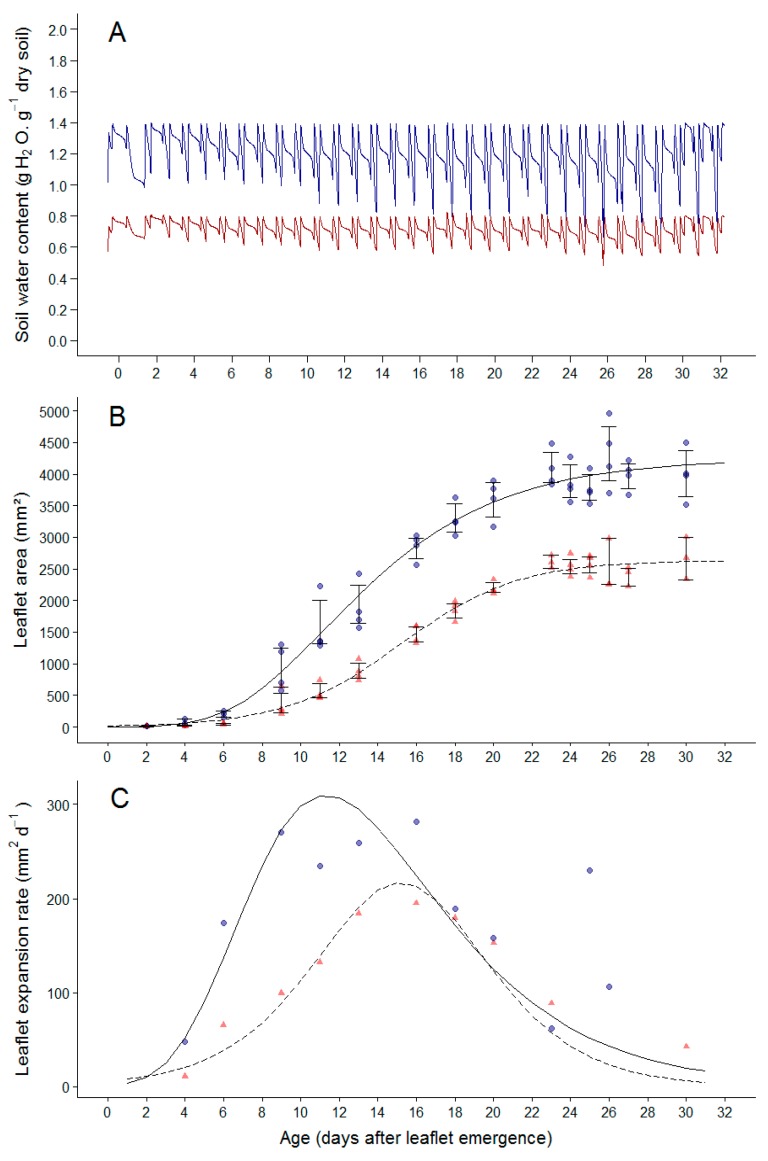
Changes with time in soil water content during experiment B (**A**) and corresponding dynamics of leaflet expansion related traits (**B**,**C**). In A, changes with time in soil water content are shown for plants in the well-watered (blue line) and water deficit treatments (red line). In B, dynamics of leaflet area are shown for leaflets two and three of the second leaf of the second sympodial unit in Wva106 plants grown in well-watered (WW) and water deficit (WD) treatments, blue circles and red triangles, respectively. Each point represents mean leaflet area of the two leaflets for an individual plant. The fitted curves are Gompertz adjustment for the WW treatment (full line) and a standard sigmoid for the WD one (dashed line, which fitted better than Gompertz). Vertical bars are standard errors around the mean leaflet area at each time point. In C, each point is the mean absolute leaf expansion rate calculated as the slope of the relationship between leaflet area and time, in Wva106 plants grown in WW and WD treatments, blue circles and red triangles, respectively. Black lines are derivative of the fitted functions shown in B for WW and WD treatments, in full lines and dashed lines, respectively.

To further dissect the effect of soil water deficit on individual leaf expansion, the dynamics of leaf expansion and cellular related traits were measured on plants grown at 1.4 g H_2_O g^−1^ dry soil referred to as well-watered (WW) treatment and at 0.8 g H_2_O g^−1^ dry soil referred to as water deficit (WD) treatment. Measurements were performed on leaflets two and three (opposite leaflets with same characteristics) on the second leaf of the second sympodial unit. The intensity of soil water deficit was stable over time for the two treatments from leaf emergence until the end of expansion (Figure 4A,B). Cellular expansion-related traits were measured on the upper epidermis of these same leaflets. These leaflets were considered as representative of the whole leaf as demonstrated before in [26].

Leaflet area followed a sigmoidal curve over time both in the WW and WD treatments (Figure 4B). In the WW condition, leaflet expansion rate first increased with time to reach a maximal value at approximately 10 days after leaflet emergence and then decreased until final leaflet area was reached, 28 days after leaf emergence (Figure 4C). For plants grown under WD treatment, leaflet expansion rate was reduced during the first phase of leaflet expansion compared to that of the WW treatment (Figure 4C). The maximal leaflet expansion rate was lower and was reached later during expansion than for the WW plants, i.e., at approximately 16 days after leaflet emergence (Figure 4C). At the end of leaflet expansion, the final area reached 2500.9 mm² in the WD treatment compared to 4032.3 mm² in the WW treatment (Figure 4B). The total duration of leaflet expansion was not affected by the WD treatment.

**Figure 5:**
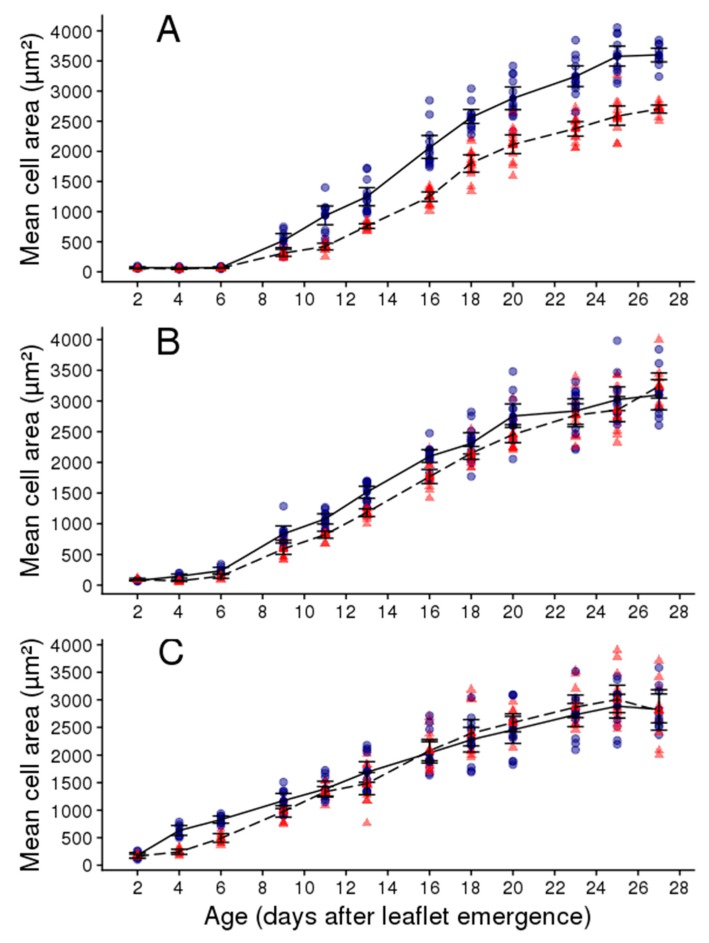
Dynamics of adaxial epidermal cell area in leaflets two and three of the second leaf of the second sympodial unit in Wva106 plants grown in WW and WD treatments, blue circles with full lines and red triangles with dashed lines, respectively. In each leaflet, cell areas were measured in three zones: At the base (**A**), in the middle (**B**), and at the tip (**C**). Each point represents mean epidermal cell area within a given zone of the leaflet (*n* = 75). The full and dashed lines link mean cell area. Vertical bars are standard errors around the mean value at each leaflet age.

**Figure 6:**
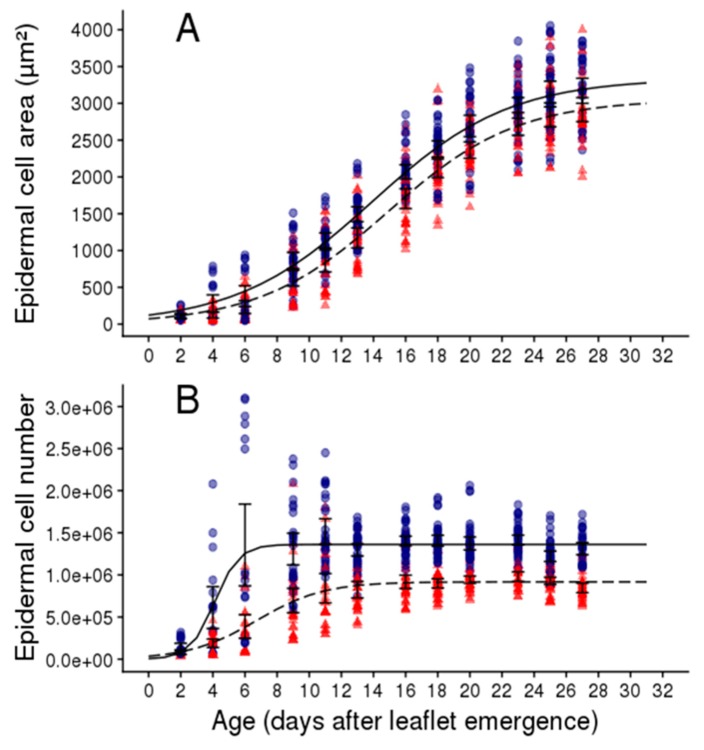
Dynamics of cell expansion (**A**) and cell production (**B**) in adaxial epidermis of leaflets two and three of the second leaf of the second sympodial unit in Wva106 plants grown in WW and WD treatments, blue circles and red triangles, respectively. Each point represents mean epidermal cell area (**A**) or mean epidermal cell number (**B**) for one leaflet. Black lines are adjusted curves with a Gompertz model for each variable in full lines for the WW and dashed lines for WD treatments, respectively. Vertical bars are standard errors around the mean value at each leaflet age.

The mean epidermal cell area was studied in three zones of the leaflets: At the base, the middle, and the tip (Figure 5). At the base of the leaflet, the mean epidermal cell area followed a sigmoidal curve over time in the two watering regimes (Figure 5A). The mean cell area was first low and remained quasi-stable during six days after leaflet emergence both in the WW and WD treatments (Figure 5A). Then, it increased with time more rapidly in the WW than in the WD treatment to reach a final value which was higher in the WW than in the WD treatment (Figure 5A). The end of cell expansion at the base of the leaflet coincided with that of whole leaflet and occurred approximately 26 days after leaflet emergence. The dynamics of cell expansion in the middle and tip part of the leaflet was similar to that observed in the basal one, but the final epidermal cell area was less affected by the WD treatment in these two zones than at the base (Figure 5B,C). In addition, the mean epidermal cell area increased more rapidly with time at the tip than at the base of the leaflet (Figure 5A,C). Reduction in leaflet expansion rate and final leaflet area observed under the WD treatment were then accompanied by a reduction in mean epidermal cell area (Figure 6A) but this was mainly due to the reduction of cell area in the basal zone of the leaflet (Figure 5).

Epidermal cell production in the whole leaflet was lower in leaflets of plants grown in the WD treatment, but the phase of cell proliferation was longer compared to the WW treatment (it lasted approximately 13 days after leaf emergence instead of six days, Figure 6B). Finally, at the end of cell proliferation, the epidermal cell number was lower in leaflets of plants grown in the WD treatment (Figure 6B). Reduction in final leaflet area caused by the WD treatment was accompanied by a reduction in cell production rate and final cell number but a lengthening of the phase with cell proliferation.

### 2.3. Soil Water Deficit Affects Both the Dynamic of Ploidy Distribution and the Final Ploidy Distribution in Leaflets

**Figure 7:**
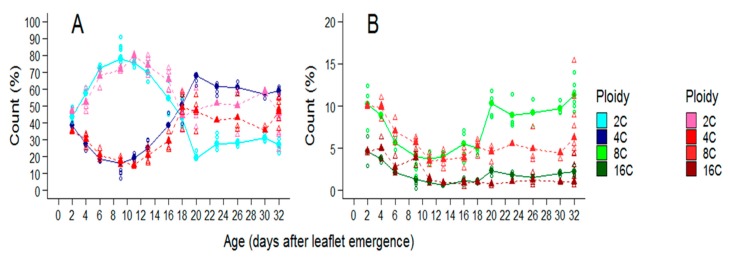
Changes with time in the proportion of 2C, 4C, 8C, and 16C cells in leaflets two and three of the second leaf of the second sympodial unit in Wva106 plants grown in WW and WD treatments. Proportions of cells in 2C and 4C are shown in (**A**), 8C and 16C are shown in (**B**), for the two treatments. Green and blue colors are used for the WW treatment, pink and red colors are used for the WD treatment (see scales on the right of the plot). At each leaf age and for both treatments, each empty point is the proportion of cells in each fraction as measured by flow cytometry within a leaflet. At each leaf age and for both treatments, each full point is the mean fraction when all flow cytometry spectra of leaflets of a same age are pulled and analyzed together.

Because soil water deficit could also affect cell ploidy and this, in turn, could impact cell size, changes with time of ploidy distribution of cells in whole leaflets were considered in plants grown in the two soil water treatments (Figure 7).

Two days after leaflet emergence, 2C cells represented the highest proportion of cells in leaflets both in WW (43.6%) and WD treatments (47.3%). The proportion of 4C cells was slightly lower, representing 38.4% and 35.1% in leaflets of plants grown in WW and WD treatments, respectively (Figure 7A). At the same age, 8C and 16C cells represented 10.3% and 4.7% of the cells, respectively, without any difference between the two treatments (Figure 7B).

During a first phase, the proportion of 2C cells in leaflets increased with time while the proportion of cells in all other three categories decreased in plants of both treatments (Figure 7A,B). For WW plants, nine days after leaflet emergence, the proportion of 2C cells was maximal and reached 78.1% whereas 4C cells represented 16.1% and the others, 4.1 and 1.3%, respectively for 8C and 16C cells (Figure 7A,B). Soil water deficit did not affect the general dynamic of ploidy distribution during the first part of leaf development, but the duration of the first phase was a bit lengthened (Figure 7A,B). The maximal proportion of 2C cells was reached 12 days after leaflet emergence and this age coincided with that of the minimal proportion of 4C cells in the leaf.

During a second phase, for WW plants, from nine days after leaflet emergence, the proportion of 2C cells decreased with time and then reached a plateau 20 days after leaflet emergence (Figure 7A). This decrease started later for the plants of the WD treatment and the plateau was reached earlier, 17 days after leaf emergence (Figure 7A). From nine days after leaflet emergence, the proportion of 4C and 8C cells increased with time and then reached a plateau 20 days after leaflet emergence for plants of the WW treatment and 17 for the WD treatment (Figure 7A,B). 

At the end of leaflet expansion, the 2C cells represented 30.9% of the cells in leaflets of plants of the WW treatment but 58.7% in plants of the WD one (Figure 7A). The 4C cells represented 56.9% of the cells in leaflets of plants of the WW treatment and 35.5% for the WD one (Figure 7A). The 8C cells represented 9.7% of the cells in leaflets of plants of the WW treatment and 4.5% in the WD one. The proportions of 16C cells remained low around 2.1 and 1%, for plants of the WW and WD treatment, respectively (Figure 7B).

## 3. Discussion

### 3.1. Different Drought Dose Responses for Growth and Development-Related Traits

The concept of drought tolerance has been defined as the ability to limit the decrease of biomass production in response to a soil water deficit [27]. This ability encompasses several morphological and physiological traits, including the sensitivity of leaf expansion to water deficit, the potential for osmotic adjustment, the stomatal sensitivity, and/or the adjustment of the root: Shoot ratio as examples [28,29]. Four of the six watering regimes applied here caused a decrease in tomato leaf dry weight in Wva106 plants, showing that below a threshold, corresponding to given soil and plant water potentials, soil water content was insufficient for maximal leaf growth and caused a reduction in leaf expansion rate. This does not mean that it was not sufficient for other vegetative related traits as shown by the stability of leaf number, leaf emergence rate, duration of leaf expansion, and specific leaf area. Such a result is not surprising per se as it was frequently reported for other plants and other drought treatments that leaf development is less affected than leaf growth in a large range of soil water deficit conditions [11,30]. In this work, we focused on growth-related traits without measuring physiological processes. Other works have revealed different responses to drought for physiological processes when compared to growth processes [31,32]. The most illustrated example is certainly that leaf growth is more sensitive to drought than photosynthesis. Moderate drought intensities indeed reduce leaf expansion but do not affect net photosynthesis as shown in *Arabidopsis thaliana* for which moderate drought treatments actually leads to a more positive carbon balance [33].

### 3.2. Drought Induced Plasticity in Cell Division and Cell Expansion in Leaves

Even if the cellular control of organ growth is under debate in the literature opposing the ‘cellular theory’ and the ‘organismal theory’ of organ growth control [34,35], the sum of cell proliferation and cell expansion responses to environmental conditions is undoubtedly reflected in the organ size response. How cellular processes underlying leaf growth plasticity are impacted by drought treatments has been investigated in many species [19,36,37]. Automated platform with automatic irrigation and automation of image analyses both at the leaf and cellular scales have been developed mainly for plants with simple leaves such as maize or *Arabidopsis thaliana* [38,39,40]. The throughput of the drought response analyses has been increased and results obtained for one or two genotypes during the 90’s have been reinforced more recently with genetic analyses on populations of recombinant inbred lines or comparing accessions [23,37]. Most of these works have reported negative impacts of drought on both final cell number and size when the drought treatment is imposed during both cell proliferation and cell expansion phases, as shown here for the tomato compound leaf. The development of framework for kinematic analyses and the observation of spatial gradients in cell division and expansion in simple leaves of monocots and dicots have increased the interest for spatial and temporal analyses of these two processes during leaf development [41]. As shown for many simple leaves in dicotyledonous, cell expansion in tomato leaflet starts earlier at the tip than at the base [42]. This reflects a gradual cessation of cell proliferation from tip to the base of these leaves. Cell size was more affected at the base of the leaflet than at the tip with an intermediate effect in the middle of the leaflet. Cell expansion was then more affected by drought in zones with a longer cell proliferation phase. The differential impact of drought stress depending on the leaf zone described here was not reported before to our knowledge.

Combining kinematic analyses at the organ and tissue scales together with nuclei observations have revealed that an important checkpoint in the regulation of the cell proliferation in response to drought is at the G1-S transition of cell cycle [17,43,44]. A blockage at the G1-S transition causes an increase in cell cycle duration and once cells pass the gate of G1-S phase, they are then capable of traversing S, G2, and M phases at the same rate as cells grown in the absence of stresses [45]. Blockage at the G1-S transition causes reduction in cell number if the duration of the cell proliferation phase is not lengthened; and subsequently, it impairs organ expansion if cell expansion is not increased for compensation. In the tomato leaflet, drought did not cause any increase in 4C cells during early development, suggesting that the decrease in cell proliferation rate, corresponding to a lengthening of cell cycle duration, is more due to a proportional increase of all cell cycle phases that a specific blockage at the G1-S transition. The duration of the cell proliferation phase in the whole leaflet was lengthened by drought in the epidermis but final epidermal cell number was decreased and was not compensated for by an increase in cell size as shown in other plant species for this type of stress [37,38,46].

### 3.3. Drought Induced Plasticity in Ploidy in Leaf Cells

During the first phase of leaf development, cells proliferate in the different leaf tissues and 4C cells measured by flow cytometry can be either cells engaged in the cell cycle just before mitosis (in the G2 phase) or endoreduplicated cells without possible distinction. In *Arabidopsis thaliana* leaves, it was proposed that endoreduplication started when cell division decelerate [47]. It is not the case here in tomato leaflet as we observed 8C and 16C cells in leaflet while cells were still actively dividing at least in the epidermis. This low proportion of cells with high level of ploidy could be specialized type of cells in other tissues than the epidermis [48]. While cells divide in the leaflet epidermis, the proportion of 2C cells increased over time in the whole leaflet and this coincided with a decrease in the proportion of 4C, 8C, and 16C cells. This decrease could be at least partly due to a “dilution” by the increasing number of cells. The proportion of 4C, 8C, and 16C cells increased afterwards and this could reflect a decrease in cell proliferation with a shift towards endoreduplication cycle for a higher proportion of cells. This shift occurred later in plants of the drought treatment than those of the well-watered ones and this coincided with a longer cell proliferation phase observed as a response to drought. Even if our result support that both mitotic cycle and endocycle coexisted in the leaf soon after its emergence, they also suggest that endoreduplication is more active after the cell proliferation phase as usually stated in the literature [49]. Increase in the proportion of 4C, 8C, and 16C cells started later in the drought treatment and stopped earlier, and as a consequence, the proportion of endoreduplicated cells was lower in leaflets of plants grown under drought treatment at the end of their development.

Taken together, our results show that cell division, expansion, and the extent of endoreduplication are all reduced in leaflets of tomato plant subjected to drought, accompanying the reduction in leaflet area and biomass. The relationships between endoreduplication, cell division, and cell expansion is unclear but our results corroborate that variation in the duration of cell proliferation (within an organ zone from tip to base in a leaflet, or the whole organ when it is affected by drought as here) could be crucial for the determination of final cell size and/or the extent of endoreduplication [50].

## 4. Materials and Methods

Two independent experiments were performed for this work. Experiment A was performed with the aim to establish dose-response of leaf growth related traits with six drought scenarios, whereas experiment B was performed to analyze the dynamics of leaf expansion related traits at two soil water contents: One well-watered treatment versus a moderate soil water deficit treatment (defined from the response curves established during experiment A).

### 4.1. Plant Material, Sowing and Seedlings Pre-Culture

147 and 210 seeds of cherry tomato plants (*Solanum lycopersicum*) line West Virginia 106 (Wva106) were sown both in experiments A and B.

During experiment A, seeds were sterilized in a solution of Barychlore (0.5 g Barychlore and 50 mL ethanol 50%) during 15 min followed by three rinses with absolute ethanol and dried under laminar flow hood during at least 15 min. Then, seeds were sown in sterilized boxes filled with a ¼ Murashige and Skoog medium (MS including vitamins, Duchefa, MO 222) with 7.5 g sucrose L^−1^ and 8 g phyto-agar L^−1^. The pH of the solution was adjusted between 5.8 and 6 with a solution of KOH 2M and the MS medium was sterilized. Boxes were set up in a growth chamber in which light was provided by a bank of cool-white fluorescent tubes and iodide discharge lamps during 16 h day^−1^ with a photosynthetic photon flux density of 200 µmol m^−2^ s^−1^ at pot height. Air vapor pressure was kept around 0.8 kPa and temperature was set at 25 °C and 20 °C during day and night periods, respectively. Boxes stayed during 20 days in the growth chamber. Then, 49 pots of 7 L were filled with soil (Klasmann, Substrat SP 15%). Pots were weighted before and after filling with soil. Soil aliquots were dried to estimate the amount of dry soil and the water content in each pot at the time of filling (Granier et al., 2006). This was used to calculate and adjust soil water content at different dates and intensities during the experiments (see below). Three tomato seedlings (from the boxes) were then put in the center of each pot and immediately irrigated with 30 mL of nutrient solution (Liquoplant rose, Plantin, dilution 4 per 1000).

In a supplementary experiment, we showed that pre-sowing in boxes had no effect on plant development compared to direct sowing in soil (not shown). Then, during experiment B, seeds were immediately sown in the same soil as described before in seventy pots. The pot size, nutrient solution, growth chamber, and conditions in the growth chamber were similar to those applied in experiment A. Seedlings were irrigated manually with 30 mL of nutrient solution twice a day for one week. After eight weeks in the growth chamber, plants were too high for further measurements and were transferred to a greenhouse with a climatic regulation close to the one used in the growth chamber (not shown).

In both experiments, plants were thinned out 10 days after transfer or direct sowing in the pots, considering developmental stage homogeneity, to keep only one plant per pot. Lateral shoots were removed and flowers were shaken three times a week during the whole experiment.

### 4.2. Automatic Adjustment of Soil Water Contents

In both experiments, soil water content was adjusted automatically pot per pot using the PHENOPSIS automatic platform in the growth chamber [26,46]. During experiment B, the automatic adjustment of soil water content was done by the PHENODYN automatic platform as soon as the plants were transferred into the greenhouse. For each drought scenario, one plant was cut and weighed weekly to adjust the target soil water content considering plant weight in the pot.

During experiment A, 42 plants were grown at a constant soil water content of 1.4 g H_2_O g^−1^ dry soil until the fifth leaf of the first sympodial unit was fully emerged, i.e., when all leaflets were unfolded. From this developmental stage, automatic irrigation was modified to reach five different “target” soil water contents which were then maintained stable over time until the end of the experiment (seven plants per regime): 0.6, 0.9, 1.2, 1.4, and 1.6 g H_2_O g^−1^ dry soil respectively (seven plants per treatment). At the same stage, seven Wva106 plants were submitted to a more severe water deficit induced by cessation of irrigation until the end of the experiment (swd). 

During experiment B, 70 plants were grown at a constant soil water content of 1.8 g H_2_O g^−1^ dry soil until 10 days after sowing. Then, plants were split into two groups, each of them with a specific soil water deficit scenario: Half of the plants remained at 1.4 gH_2_O g^−1^ dry soil (well-watered condition) and others at 0.8 gH_2_O g^−1^ dry soil (moderate soil water deficit condition as shown from results of Experiment A).

### 4.3. Measurements of Leaf Growth Related Traits for Dose Response Analyses

During experiment A, for each of the six drought scenarios, leaf production-related traits were measured over time (three times a week) on each individual plant by counting the number of visible leaves on the first sympodial unit. The final number of leaves of the first sympodial unit was the number of leaves when it did not change anymore between two consecutive dates. Leaf emergence rate was calculated as the slope of the relationship between the number of leaves and time after the first leaf emergence.

When all compound leaves of the first sympodial unit had reached their final size (as checked by daily measurements of the length of the last emerged leaflet), each leaf was cut at the base of its rachis and scanned. Areas of individual leaflets were measured on the leaf scans with the ImageJ image analysis software (Wayne Rasband, National Institutes of Health, USA). For each compound leaf, whole leaf area was calculated as the sum of its leaflet areas. The first sympodial unit had between 10 and 13 leaves depending on treatments and plants within a treatment. Final leaf area varied between the first leaf ranks but not for leaves seven to 13 which had the same final leaf area (as previously shown in [46]). For measurements of leaf growth and leaf expansion related traits presented below, leaves seven to 10 which were present on each individual plant and spent all their development during the targeted watering scenario were considered. Mean final leaf area per plant was calculated averaging areas of leaves seven to 10. Individual laminas of leaves seven to 10, i.e., leaves without their rachis and petioles, were then put in individual paper bags and left in an oven at 60 °C for five days. Individual leaf dry weight was then measured on a balance and mean leaf dry weight was calculated averaging dry weights of leaves seven to 10 of a plant. First, specific leaf area was calculated as the ratio between leaf area and dry weight considering leaves individually. Then, mean specific leaf area per plant was calculated averaging specific leaf areas of leaves seven to 10 of a same plant.

### 4.4. Measurements of Leaf Growth Related Traits for Dynamic Analyses

During experiment B, a set of leaf growth-related traits were measured at the leaflet scale, cellular scale and subcellular scale, three times a week on the second leaf of the second sympodial unit from their emergence until the end of their growth. This was done on both drought scenarios: The well-watered and soil water deficit treatments. As previously shown in [46], leaflets of a same leaf are equivalent to each other. So, we considered here, that leaflet growth-related traits measured on leaflets two and three of the second leaf of the second sympodial unit summarized whole leaf growth-related traits of this leaf which spent all its development during stable soil water content conditions.

At the leaflet scale, leaflets two and three of the second leaf of the second sympodial unit were cut each two days during the first week following their emergence. They were scanned for area measurements by ImageJ. After this first week, they were large and flat enough for non-destructive measurements, so pictures were taken on the same leaflets over time three times a week and leaflet areas was determined by Image J on these pictures. Leaflet areas were plotted against time.

At the cellular scale, adaxial epidermal imprints were obtained by drying off a translucent varnish coat spread on the adaxial side of both leaflets two and three of the second leaf of the second sympodial unit. These leaflets were those that were cut for leaflet area determination just after their emergence and this protocol was followed during one week (see before). The imprint was peeled off and immediately stuck on a microscope slide with one sided adhesive. After one week, leaflets were large and flat enough for non-destructive analyses of their epidermal cells. Imprints were done with a toothpaste (Coltene President Light Body Surface Activated, Patterson Dental, Detroit MI) as detailed in [51]. After drying for one minute the toothpaste was delicately removed from the leaflet surface. Nail varnish was then spread on the toothpaste, peeled off one minute later, and immediately stuck on a microscope slide with one sided adhesive. Whatever the method (nail varnish which was destructive or toothpaste which was non-destructive), all imprints were placed under a microscope (Leitz DM RB; Leica, Wetzlar, Germany) coupled to the ImageJ image analysis software. For each leaflet, epidermal cell area was obtained by drawing cells in nine zones of the leaflet: three at the base, three in the middle, and three at the tip. The mean epidermal cell number per leaflet was calculated as the ratio between mean leaflet area and mean leaflet epidermal cell area (considering all three zones).

At the subcellular scale, just before leaf scan, discs of leaflets were harvested at the base, middle and tip of the leaflet on the left side of the midvein, using a punch of 8 mm diameter. Discs were immediately put in a 2 mL Eppendorf and frozen in liquid nitrogen. Samples were then stored at −80 °C until flow cytometry measurements. Frozen disks were chopped with a razor blade and incubated in 200 µL extraction buffer for two minutes. Extracted nuclei were fixed with 200 µL of 70% ethanol for 2 minutes and colored with 800 µL DAPI. The solutions were filtered to eliminate all structures with diameter higher than 30 µm. Remaining solutions were analyzed by flow cytometry with a C6 BD Accuri system. All reagents were obtained from BD Biosciences. Ploidy histograms were quantitatively analyzed with the R software, after manual treatment to remove noise. Peaks of cells with nuclei in 2C, 4C, and 8C were first manually identified and positioned with nuclei extracted from young tomato leaves with a high proportion of dividing cells. Positions of peaks of cells with nuclei in 16C were deduced from other peak positions assuming that DNA content was additive. Ploidy histograms were equivalent between different zones of a leaflet and repetitions. Then, they were pooled according to leaflet age and soil water content treatment.

### 4.5. Fitted Curves of Leaflet Expansion Related Traits During Experiment B

Data of leaf area, cell area and cell number were plotted against time and sigmoid curves were fitted using either the logistic model (Equation (1)) or the Gompertz model (Equation (2)):

𝑦 = 𝑎/[1 + exp{−([−exp{(𝑡 − 𝑡0/𝑏}] + 𝜀(1)

𝑦 = 𝑎 exp[−exp{−(𝑡 − 𝑡0)/𝑏}] + 𝜀(2)

where *a* is the upper asymptote, 𝑡0 is time at the inflection point, *b* is the characteristic duration and 𝜀 is the residual term distributed by a normal law centered in 0. Parameters *a*, 𝑡0, and *b* were estimated using the Gauss-Newton algorithm.

### 4.6. Statistical Analyses

Statistical significance of trait variation was tested by Wilcoxon-Mann-Whitney non parametric test. The signification level was fixed at α = 5%. Risk level of the test was evaluated by the *p*-value.
